# Targeted next-generation sequencing identification of mutations in patients with disorders of sex development

**DOI:** 10.1186/s12881-016-0286-2

**Published:** 2016-03-15

**Authors:** Yanling Dong, Yuting Yi, Hong Yao, Ziying Yang, Huamei Hu, Jiucheng Liu, Changxin Gao, Ming Zhang, Liying Zhou, Asan, Xin Yi, Zhiqing Liang

**Affiliations:** Department of Obstetrics & Gynecology, Southwest Hospital, Third Military Medical University, Chongqing, China; Binhai Genomics Institute, BGI-Tianjin, Tianjin, China; BGI-Shenzhen, Shenzhen, China; Tianjin Enterprise Key Laboratory of Clinical Molecular Diagnostic, Tianjin, China

**Keywords:** Disorders of sex development, Targeted next-generation sequencing, Novel mutation

## Abstract

**Background:**

The identification of causative mutations is important for treatment decisions and genetic counseling of patients with disorders of sex development (DSD). Here, we designed a new assay based on targeted next-generation sequencing (NGS) to diagnose these genetically heterogeneous disorders.

**Methods:**

All coding regions and flanking sequences of 219 genes implicated in DSD were designed to be included on a panel. A total of 45 samples were used for sex chromosome dosage validation by targeted sequencing using the NGS platform. Among these, 21 samples were processed to find the causative mutation.

**Results:**

The sex chromosome dosages of all 45 samples in this assay were concordant with their corresponding karyotyping results. Among the 21 DSD patients, a total of 11 mutations in *SRY*, *NR0B1*, *AR*, *CYP17A1*, *GK*, *CHD7*, and *SRD5A2* were identified, including five single nucleotide variants, three InDels, one in-frame duplication, one *SRY*-positive 46,XX, and one gross duplication with an estimated size of more than 427,038 bp containing *NR0B1* and *GK*. We also identified six novel mutations: c.230_231insA in *SRY*, c.7389delA in *CHD7*, c.273C>G in *NR0B1*, and c.2158G>A, c.1825A>G, and c.2057_2065dupTGTGTGCTG in *AR*.

**Conclusions:**

Our assay was able to make a genetic diagnosis for eight DSD patients (38.1 %), and identified variants of uncertain clinical significance in the other three cases (14.3 %). Targeted NGS is therefore a comprehensive and efficient method to diagnose DSD. This work also expands the pathogenic mutation spectrum of DSD.

**Electronic supplementary material:**

The online version of this article (doi:10.1186/s12881-016-0286-2) contains supplementary material, which is available to authorized users.

## Background

Disorders of sex development (DSD) are a group of rare conditions involving atypical chromosomal, gonadal, or anatomical sex development. The prevalence of DSD has been estimated to be about 1/4500 live births [1]. According to karyotype, DSD can be classified into sex chromosome DSD, 46,XX DSD, and 46,XY DSD [2], and correct classification is critical for gender assignment, genital surgery, and lifelong care [3]. Thus, accurate genetic testing and the understanding of genotype–phenotype correlations will help refine the diagnosis and management of DSD.

The genetic causes of DSD are heterogeneous [4], with more than 60 genes reported to associate with sex determination (gonadal dysgenesis, testicular and ovotesticular DSD), sex differentiation (*e.g.*, steroid synthesis/receptors), and hypogonadism [5, 6]. Moreover, the genetic changes that occur in DSD are highly complex, including single nucleotide variants (SNVs), small insertions and deletions (InDels), copy number variations (CNVs), *SRY*-positivity in XX individuals, and sex chromosome abnormalities.

Current genetic testing using Sanger sequencing of candidate genes is both inefficient and time-consuming. Using this method, only 20 % of DSD cases can be diagnosed, leaving most cases of gonadal dysgenesis undiagnosed at the genetic level [5]. In contrast, next-generation sequencing (NGS) can provide high-throughput, accurate screening of multiple genes and different types of mutations in a highly efficient manner. Indeed, NGS has been used to diagnose affected individuals of numerous types of disorders affecting multiple genes [7].

A previous pilot testing for DSD involving 35 known DSD genes associated with sex determination and sex differentiation identified genetic causes in two out of seven patients [8]. This relatively low detection rate represented the small gene set in the panel, so expanding the number of candidate genes to several hundred would potentially improve this. In the present study, we performed the genetic testing of 219 DSD-associated genes in a cohort of DSD patients using targeted NGS to expand the DSD spectrum of pathogenic mutations.

## Methods

### Patients and other subjects

Twenty-one Chinese Han probands with DSD (DSD01–DSD21) were included in this study. Their phenotypes and clinical findings are described in Table 1. Two patients had siblings affected with the same phenotypes. All patients were diagnosed based on ESPE/LWPES guidelines [9]. An Asian genome (YH) was used to assess the detection power of our assay [10]. An additional 21 subjects (C01–C21) were recruited for sex chromosome dosage validation, including 17 unaffected individuals with known karyotypes and four individuals with 47,XXY or 45,XO. The study was approved by the Institutional Review Board of BGI, and informed consent was obtained from all patients before blood sampling.

**Table 1:** Phenotypic description and previous clinical findings

SampleID	External genitalia	Anatomy	Gonads	Additional clinical findings	Other diagnostic tests
DSD01	female	no uterus	streak gonad on left side; no gonad on right	primary amenorrhea; mixed germ cell tumor on right pelvis; tall stature; breast tannerIII	46,XY;elevated FSH、Testosterone and LH;normal PRL E2
DSD02-1	female	no uterus	bilateral ovary hypoplasia	Primary amenorrhea; Bilateral ovarian yolk sac tumor	46,XY; Familia;elevated FSH,Testosterone and LH;normal PRL E2
DSD02-2	female	no uterus	bila. likely fallopian tube tissue	primary amenorrhea; serous cystoma on right gonad; dysgerminoma on left gonad	46,XY; Familia;elevated FSH,Testosterone and LH;normal PRL E2
DSD03	male	hypospadia	no records	no records	46,XY; *SRY* negative
DSD04	female	small uterus	normal gonad tissue	primary amenorrhea; short stature; Dysplastic ears	46,XX
DSD05	male	hypospadia; Genital hypoplasia	two abdominal testes, normal testicular tissue	no records	46,XX; *SRY* negative
DSD06	female	no uterus;1/3vagina present; Fallopian tubes present	ovary; Fallopian tube	primary amenorrhea	46,XX; normal female hormonal profile
DSD07	male	hypospadia	no records	no records	46,XY; aCGH
DSD08	male; micropenis	Genital hypoplasia; hypospadia; 2 cm phallus	two abdominal testes, normal testicular tissue	no records	46,XX
DSD09-1	male; micropenis	Genital hypoplasia; hypospadia; 1.8cmphallus, small scrotum	normal testicular tissue	no records	46,XY; Familia
DSD09-2	male	Genital hypoplasia; hypospadia	normal testicular tissue	no records	46,XY; Familia
DSD10	male	Genital hypoplasia; cavernosa	no testes tissue;likely uterus tissue on pelvis	no records	46,XX; *SRY* negative
DSD11	male; micropenis	Fallopian tubes and small uterus; hypospadia; 1.5 cm phallus, small scrotum	likely uterus tissue on pelvis	no records	46,XX; *SRY* negative
DSD12	female	small uterus	normal gonad tissue	primary amenorrhea	46,XX
DSD13	female	no uterus	bila.streak gonad	primary amenorrhea	46,XY; elevated testosterone
DSD14	female	no uterus	ovotestis with Fallopian tube	primary amenorrhea	46,XY; elevated testosterone; FISH
DSD15	ambiguous (raised female)	small uterus, Genital hypoplasia	streak gonad on right side; no gonad on left	primary amenorrhea;	46,XY; deceased E2;elevated FSH
DSD16	female (clitorism)	no uterus	none found by ultrasound	primary amenorrhea	46,XY; elevated testosterone; FISH
DSD17	female	no uterus	none found by ultrasound	primary amenorrhea	46,XY
DSD18	female (labia minora hypertrophy)	no uterus; blind vagina	Partial gonadal dysgenesis	primary amenorrhea	46,XY; elevated testosterone; FISH
DSD19	male	Genital hypoplasia; hypospadia	no uterus;no ovary; two abdominal testes; normal testicular tissue	no records	46,XX; *SRY* negative; decreased E2, P, PRL, FISH; no testosterone
DSD20	female (clitorism)	no uterus	none found by ultrasound	primary amenorrhea	46,XY; elevated testosterone; FISH; LH
DSD21	ambiguous (raised female)	no uterus, ovary	none found by ultrasound	primary amenorrhea	46,XY

### Disease selection and panel design

The DSD testing panel was designed to contain previously reported clinically associated genes [11], and genes associated with sex determination, sex differentiation, and hypogonadism from the Online Mendelian Inheritance in Man (OMIM) database [12], including a subset of genes for syndromes with sex development in OMIM. An additional set of unique genes with an average 10 Mb intermediate distance on X and Y chromosomes were included in the panel to estimate sex chromosome dosage (Additional file 1). Exons and flanking sequences of the above genes were targeted in the primary target regions.

Manufacture of the DSD panel was based on the NimbleGen SeqCap EZ Choice Library (Roche). Primary target regions of HG19 NCBI Build 37.1/GRCh37 were submitted online and preprocessed before probe selection. Regions smaller than 100 bp in size were padded to 100 bp from the center to allow for more probe candidates, and overlapping regions were subsequently merged. Probes were selected according to the manufacturer’s standard protocols with preferred close matches of 3 and maximum close matches of 5 to minimize cross-hybridization. A total of 2972 exons, and 1,078,042 bases of 219 genes were captured and sequenced in this study.

### Targeted genomic capture and next-generation sequencing

Targeted genomic capture and NGS was performed as previously described [13]. Between 20 and 30 libraries were pooled and hybridized with one customized DSD panel. The captured products were sequenced using the Hiseq2500 system (Illumina, San Diego, CA), and 0.5 G raw data were obtained for each sample.

### Alignment and variant detection

Raw image analysis, base-calling, and error rate analysis were carried out on local computer clusters using the Off-Line Base caller (Illumina, v1.9.4) with default parameters. Adaptor-contaminated reads were discarded, then reads were separated into samples according to their unique indices. For each sample, low quality reads were removed, and clean reads were mapped to the Human GRCh37/hg19 assembly using Burrows–Wheeler Aligner, v0.6.2-r126 [14]. Variant calling (SNVs and InDels) was conducted using the Genome Analysis Tool kit, v3.2-2 [15].

### Sex chromosome dosage, CNVs, and 46,XX *SRY* positive analysis

In the GC-bias correction process, the following sequential steps were taken: the GC count was determined using 30-bp sliding windows with 25-bp step sizes. Regions shorter than 30 bp were padded equally from both ends to reach 30 bp. Then the GC content and read depth were obtained for each window. Smoothing LOWESS regression was applied to the GC content and depth in each window of all chromosomes for each sample. The average depth of all windows was calculated as a baseline. Then, the GC-bias correction factor was calculated as the LOWESS regression value divided by the baseline. Finally the depth of each window was corrected by the correction factor according to its GC ratio.

**Fig. 1:**
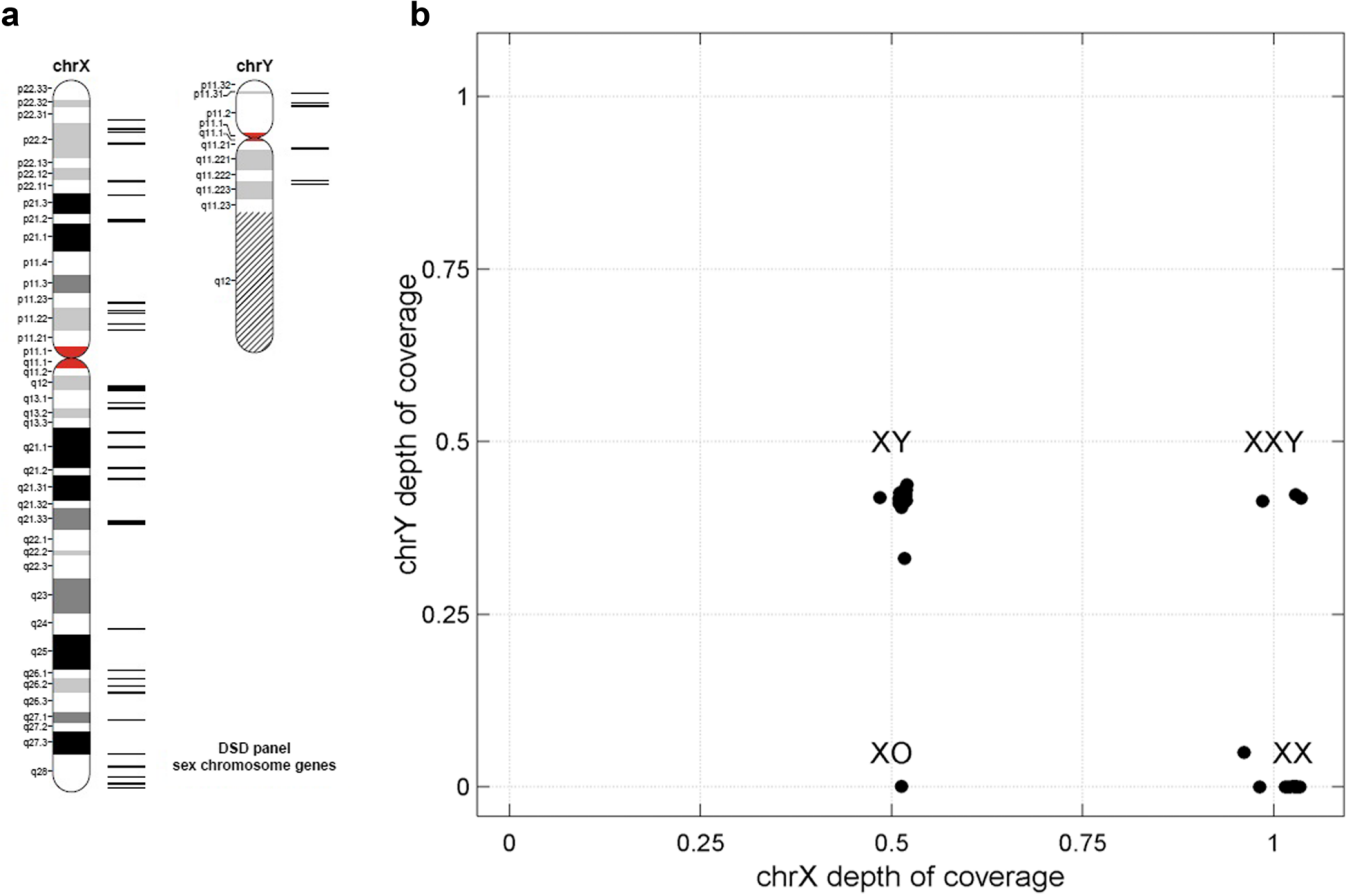
Sex chromosome dosage analysis. **a** Distribution of the target genes on sex chromosomes. Bars on the right of the ideogram indicate the location of the sex genes; thicker bars represent more genes. **b** Sex chromosome dosage distribution. Samples with karyotype XX (n = 18), XY (n = 24), XXY (n = 3) or XO (n = 1) were grouped as four clusters

Forty uniquely mapping genes on chromosome X and six uniquely mapping genes on chromosome Y were used to analyze sex chromosome dosage based on the mapping files (Fig. 1a). After the GC correction process, two steps were taken: 1) the average depth of the windows of all autosomes (ATO) was calculated as a new baseline, excluding the extreme values; and 2) the mean depth of X and Y chromosome windows was normalized to the baseline to generate chrX/chrATO and chrY/chrATO.

CNV examination within the captured regions was similar to sex chromosome dosage analysis. After GC-bias correction, the coefficient of the recalibrated depth of each window was calculated and the depth distribution profile was obtained. Subsequently, samples of low quality (mean correlation coefficient of all chromosomes <0.6, or more than four chromosomes with correlation coefficient values <4/5 of the mean value of other samples) were excluded from further analysis. Batch calibration was performed on the GC-calibrated depth of each window by negative binomial distribution fitting. Then, the hidden Markov model was implemented to estimate the copy number of each window. Regions with more than five continuous supporting windows were selected as CNV candidates.

To avoid the impact of homologous regions between sex chromosomes, samples identified as XX were realigned to the chrY-free reference sequence prior to variant calling. To detect *SRY*-positive variants in 46,XX DSD patients, unmapped pair-end reads were extracted and mapped to the reference sequence of chrY. Sequence depth and coverage of *SRY* was determined from the final mapping file.

### Confirmation of candidate variants

Each variant identified by NGS was verified by another method in the patient and available family members. In brief, SNVs and small InDels were confirmed by Sanger sequencing; CNVs were confirmed by quantitative polymerase chain reaction (qPCR); and 46,XX with *SRY*-positivity by a PCR assay. Additionally, the possible outcome of non-synonymous SNVs was evaluated by the CONsensus DELeteriousness score (Condel) that combines SIFT and Polyphen2 [16, 17] with a conservation score of phyloP.

## Results

### Evaluation of quality metrics

To validate the performance of the NGS panel (including the data analysis pipeline), we assessed key quality metrics using the YH genome as reference material. As described previously [18], we found that a sequence depth of >200× provided a genotype calling sensitivity of 98.73 % (>50× depth) with an accuracy rate of 99.43 % (Additional file 2). We also observed high reproducibility between experiments (Additional file 3). Sensitivity, specificity, and accuracy were all >99.50 % for SNV calling compared with the published genotyping data for targeted regions (Additional file 3). These results demonstrate the reliability of the experimental approach as well as the data analysis pipeline of the NGS panel.

After aligning reads to the reference human genome, the average sequencing depth for the other 44 samples on the targeted regions was 359 ± 81×, while 98.06 % of targeted regions were covered by 20 or more reads, indicating the high quality of the data (Additional file 4).

### Validation of sex chromosome dosage

In routine clinical practice, karyotype analysis is necessary for DSD diagnosis, which is essential for understanding the clinical significance of the detected variants. We used 45 samples to assess the detection reliability of sex chromosome dosage in our assay, including 17 with XX karyotypes, 24 with XY, three with XXY, and one with XO. For this purpose, the normalized depths for chromosomes X and Y (chrX/chrATO, chrY/chrATO) were calculated and independently examined for each sample. The mean normalized coverage was 0.5134 ± 0.0068 for one copy of chrX, 1.0188 ± 0.0186 for two copies of chrX, 0.4160 ± 0.0186 for one copy of chrY, and 0.0031 ± 0.0114 for null chrY (Fig. 1b, Additional file 5). Thus, samples with karyotypes of XX, XY, XXY, or XO were grouped as four clusters. The sex chromosome dosages of all 45 samples were consistent with karyotyping results.

### Mutation detection and analysis

To identify the DSD-causative mutation in each patient, we selected rare SNVs or InDels by four steps of filtering: 1) basic filtering: variants with insufficient sequence coverage (sequencing depth <8 x coverage and Phred-like quality score <30 were ruled out; 2) frequency filtering: SNVs or InDels with allelic frequencies >0.05 in the 1000 Genomes Project dataset [19] and proprietary exome sequencing dataset were excluded; 3) function region filtering: variants in the intron or untranslated region were discarded, with the exception of splice site mutations or variants recorded in the Human Gene Mutation Database; and 4) clinical phenotype filtering: pedigree co-segregation of disease phenotypes were considered to confirm the causality of the DSD variant.

Among the 21 DSD probands, 11 likely causative mutations in seven genes were identified in 11 patients (52.4 %), including 6/21 (28.6 %) with reported pathogenic findings, 2/21 (9.5 %) with likely pathogenic findings, and 3/21 (14.3 %) with variants of unknown clinical significance (VUS) according to American College of Medical Genetics and Genomics guidelines for the interpretation of sequence variants [20]. According to the proposed classification of DSD causes, we made a molecular genetic diagnosis for nine of 13 (69.2 %) 46,XY DSD patients, and two of eight (25 %) 46,XX patients.

**Fig. 2:**
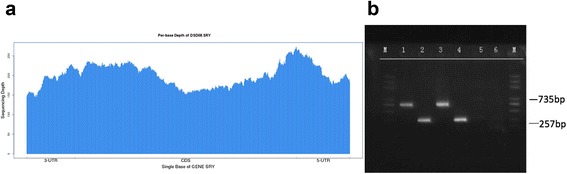
46,XX *SRY*-positive was identified in DSD08 in this assay. **a** The entire *SRY* gene was covered with a depth of more than 100× in the 46,XX sample through targeted NGS testing. **b** NGS results were confirmed by PCR assay. Two primers were designed for this test, one covered the whole *SRY* gene with a product of 735 bp, while the other covered the first 200 bp with a product of 257 bp. Lanes 1 and 2: subjects; lanes 3 and 4: *SRY*-normal samples; lanes 5 and 6: *SRY*-negative samples

Among the 11 mutations, a duplication including *NR0B1* and *GK* (approximately ChrX:30322539-30749577), c.297 + 2T>C in *CYP17A1*, and c.2359C>T (p.Arg787*) and c.174_175insTAG (p.Gln59*) in *AR* have previously been associated with 46,XY DSD. Moreover, this NGS method detected *SRY*-positivity in a 46,XX testicular DSD patient, which was subsequently confirmed by PCR assay (Fig. 2).

**Fig. 3:**
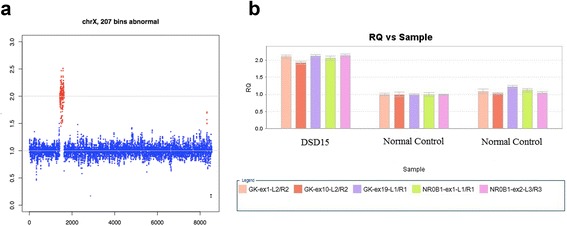
A Duplication involving *NR0B1* and *GK* was identified in patient DSD15 with 46,XY DSD. **a** CNV analysis of the subject. Blue spots represent the normal chromosome region with one copy, red spots represent the 207 abnormal bins with two copies. **b** qPCR validation. The quantity of *NR0B1* and *GK* in the subject is comparable to the normal control

We also identified six novel candidate mutations in six patients. A hemizygous c.230_231insA mutation in *SRY* was identified in a female patient with 46,XY complete gonadal dysgenesis (DSD01), and a heterozygous c.7389delA mutation in *CHD7* was found in a woman with a small uterus, primary amenorrhea, short stature, and dysplastic ears (DSD04). Both mutations were *de novo* and were predicted to significantly truncate the coded proteins (Table 2). Additionally, a hemizygous c.273C>G (p.Tyr91*) mutation in *NR0B1* was detected in a 46,XY male with hypospadias (DSD07), which was similar to a previously reported change in the same nucleotide from C to A leading to an identical amino acid change (p.Tyr91*) [21]. The remaining two missense variants and one in-frame duplication in *AR* were c.2158G>A (p.Ala720Thr), c.1825A>G (p.Arg609Gly), and c.2057_2065dupTGTGTGCTG (p.Val686_Ala688dup), respectively. The two missense mutations were predicted to change conserved sites by PhyloP and show deleterious effects by Condel. The c.2158G>A *AR* mutation was detected in two brothers (DSD09-1, DSD09-2), and had been inherited from their mother. None of these six mutations were found in the 1000 Genomes Project dataset or the in-house database consisting of 1092 Chinese Han normal controls, so were highly likely to be causative of disease (Table 2). All mutations were validated by Sanger sequencing or qPCR (Fig. 3, Additional files 6 and 7).

**Table 2:** Deleterious variant identification in DSD patients

Sample	Gene	Transcript	Nucleotide change	Protein change	Zygosity	Novel	MAD^a^/R^b^	Condel	phyloP	Origin	Interpretation
(1) Mutations identified in 46,XY DSD cases											
DSD01	*SRY*	NM_003140.1	c.230_231insA	p.Lys77fs*27	Hem	novel	127/1	.	.	*de novo*	Likely pathogenic
DSD07	*NR0B1*	NM_000475.4	c.273C>G	p.Tyr91*	Hem	novel	68/0.83	.	.	*mat*	Pathogenic
DSD09-1	*AR*	NM_000044.3	c.2158G>A	p.Ala720Thr	Hem	novel	154/1	D^c^	5.094	*mat*	VUS^e^
DSD09-2	*AR*	NM_000044.3	c.2158G>A	p.Ala720Thr	Hem	novel	131/0.98	D	5.094	*mat*	VUS
DSD13	*CYP17A1*	NM_000102.3	c.297 + 2T>C	.	Hom	reported [28]	189/0.99	.	.	ND^d^	Pathogenic
DSD14	*AR*	NM_000044.3	c.2359C>T	p.Arg787*	Hem	reported [29]	142/0.99	.	.	ND	Pathogenic
DSD15	*NR0B1;GK*	NM_000475.4; NM_000167.5	Duplication containing Chr X: 30322539-30749577	.	Het	reported [25, 26]	.	.	.	ND	Pathogenic
DSD17	*AR*	NM_000044.3	c.174_175insTAGCAGCAGCAGCAG	p.Gln59*	Hem	reported [30]	49/0.96	.	.	*mat*	Pathogenic
DSD18	*AR*	NM_000044.3	c.1825A>G	p.Arg609Gly	Hem	novel	126/1	D	1.333	*mat*	VUS
DSD20	*AR*	NM_000044.3	c.2057_2065dupTGTGTGCTG	p.Val686_Ala688dup	Hem	novel	110/0.98	.	.	*mat*	VUS
(2) Mutations identified in 46,XX DSD cases											
DSD04	*CHD7*	NM_017780.3	c.7389delA	p.K2464Sfs*39	Het	novel	138/0.5	.	.	*de novo*	Likely pathogenic
DSD08	*SRY*	NM_003140.1	.	Positive	Hem	reported [31]	.	.	.	*de novo*	Pathogenic
(3) Only one Mutation identified											
DSD02-1	*SRD5A2*	NM_000348.3	c.737G>A	p.Arg246Gln	Het	reported [32]	70/0.48	.	.	*mat*	Pathogenic
DSD21	*SRD5A2*	NM_000348.3	c.680G>A	p.Arg227Gln	Het	reported [33]	102/0.43	.	.	*mat*	Pathogenic

^a^minor allele depth; ^b^ratio of minor allele depth to total allele depth; ^c^deleterious; ^d^not determined; ^e^variant of unknown significance

Multiple variants were identified in 10 additional patients, but none showed consistency with the phenotype or the inheritance of the patients (Additional file 8). In these cases, disorders might be caused by other genetic factors that remain to be discovered.

## Discussion

In this study, we developed a customized panel for testing sex chromosome dosage changes and mutations of DSD genes using NGS-based targeted sequencing technology. Sex chromosome status is crucial for the clinical and molecular diagnosis of DSD because dosage changes of sex chromosomes are accountable for about 20 % of DSD [5] and are important in the interpretation of other types of DSD. Moreover, 46,XY DSD and 46,XX DSD inheritance is complicated because it can be caused by the same gene but result in opposite effects (function gain versus loss). In this study, all 45 patients tested by our assay showed sex chromosome dosage consistent with karyotyping results. Therefore, our assay is capable of distinguishing normal XX or XY, copy number gain, or loss of sex chromosomes.

Among the 21 patients with an unclear genetic diagnosis, we identified causative mutations in nine of 13 families with 46,XY DSD, and two of eight families with 46,XX DSD. A previous study using whole exome sequencing to diagnose 46,XY DSD identified genetic causes in 35 % (14/40) of cases, as well as six VUS variants (15 %) [8]. Thus, our assay outperforms other studies, and is consistent with exome sequencing in the diagnosis of these conditions because of the comprehensive collection of DSD-associated genes included in the panel. Unfortunately, the mutations identified in our study were limited to several common DSD genes as a result of the small sample size.

We identified six novel mutations, which expands our understanding of the mutation spectrum of DSD. A novel deletion in *CHD7* was detected in DSD04, in addition to the identification of a frame-shift mutation in *SRY* and a nonsense mutation in *NR0B1*. Autosomal dominant inheritance of truncated mutations in *CHD7* have been known to cause CHARGE syndrome [22], which is difficult to diagnose from phenotypes because of the high heterogeneity with other conditions. In the case of DSD04, the phenotype resembles that of CHARGE syndrome, so it is very likely that the novel deletion in *CHD7* is the causative mutation. In another patient, we found three previously unreported variants, p.Ala720Thr, p.Arg609Gly, and p.Val686_Ala688dup, in the DNA binding and ligand binding domains of *AR* that are likely to be causative of DSD. Variants in *AR* are known causes of androgen insensitivity syndrome (AIS) [23]. Over 500 different *AR* mutations, localized mainly in regions encoding DNA binding (AA:558–624) and ligand binding domains (AA:672–920) [23, 24], from more than 850 patients with AIS have been recorded in the Androgen Receptor Gene Mutations Database.

In patient DSD15 with ambiguous external genitalia, a small uterus, streak gonads on the right hand side and no gonads on the left, and primary amenorrhea, we found a gross duplication estimated to exceed 427,038 bp and involving *NR0B1*, *CXorf21*, and *GK*, although only 4517 bp was captured in the assay (Fig. 3a). *NR0B1* is considered to be an anti-testis gene responsible for gonadal dysgenesis, and loss-of-function mutations in this gene are accountable for congenital adrenal hypoplasia and hypogonadotropic hypogonadism. In contrast, a duplication including a 160-kb minimal common region containing *MAGEB* and *NR0B1* can cause sex reversal [25–27]. The smallest CNV segment identified by our assay was ~130 bp, suggesting that CNVs less than 130 bp in length could be missed. However, exons <130 bp can be identified by manually comparing the exon sequencing depth with other samples in the same batch. And the other CNVs with both less than 130 bp and uncovering an exon is likely to be missed. Another limitation of our analysis is that the precise breakpoint of CNVs cannot be precisely determined by our assay, and could be anywhere between the terminal base within the CNV and the adjacent base in the non-CNV.

## Conclusions

In summary, our findings show that testing a panel of genes associated with DSD can achieve a precise clinical diagnosis of the disease for phenotypically or genetically heterogeneous DSD. Our approach made a clear genetic diagnosis in eight patients (38.1 %) and identified VUS in the three other cases (14.3 %). NGS-based targeted sequencing using our assay is therefore a promising technique to improve the detection rate of DSD, which would assist clinicians in differential diagnosis, genetic counseling, and timely treatment for affected individuals.

## Additional files

Additional file 1: Genes associated with disorders of sex development (DSD). (XLSX 25 kb) Additional file 2: Quality metrics generated using YH genome sequencing data. (DOC 18.4 kb) Additional file 3: SNV validation of the YH sample. (XLSX 12.8 kb) Additional file 4: Sequencing quality statistics of all samples. (XLSX 14.4 kb) Additional file 5: Sex chromosome dosage analysis. (XLSX 14.1 kb) Additional file 6: Primers used for candidate variant validation. (XLSX 17.1 kb) Additional file 7: Sanger sequencing of candidate SNVs and Indels. (DOC 417 kb) Additional file 8: Number of candidate variants in 23 patients after filtering. (XLSX 13.9 kb)

## Abbreviations

CNV: copy number variation
DSD: disorders of sex development
Indels: small insertion and deletions
NGS: next-generation sequencing
OMIM: Online Mendelian Inheritance in Man
SNV: single nucleotide variant
