# Supplementary material for: Targeted next-generation sequencing identification of mutations in patients with disorders of sex development
Source: BMC Med Genet. 2016 Mar 15;17:23. doi: 10.1186/s12881-016-0286-2 (PMC4791760; doi:10.1186/s12881-016-0286-2)
Supplement: Additional file 2: — Quality metrics generated using YH genome sequencing data. (DOC 18.4 kb) [file 12881_2016_286_MOESM2_ESM.doc]

**Additional file 2.Quality metrics generated using YH genome sequencing data**
