# Supplementary material for: Targeted next-generation sequencing identification of mutations in patients with disorders of sex development
Source: BMC Med Genet. 2016 Mar 15;17:23. doi: 10.1186/s12881-016-0286-2 (PMC4791760; doi:10.1186/s12881-016-0286-2)

**Additional file 7.Sanger sequencing of candidate SNVs and Indels.**

Part1 Sanger sequencing results of samples identified with novel mutations

1.DSD01, *SRY*,c.230_231insA,*Hem*


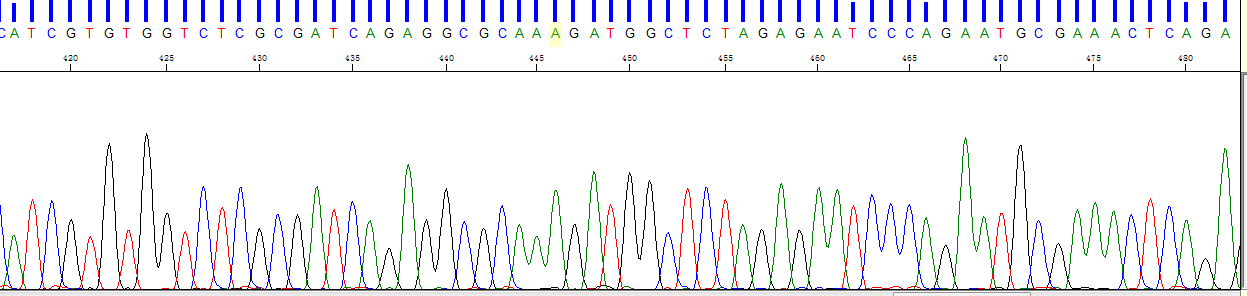


DSD01-father, *SRY*,c.230_231insA,normal


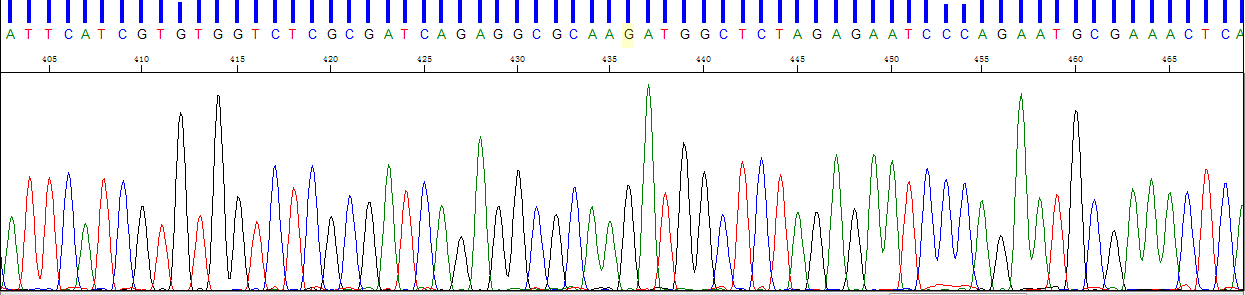


DSD01-brother, *SRY*,c.230_231insA,normal


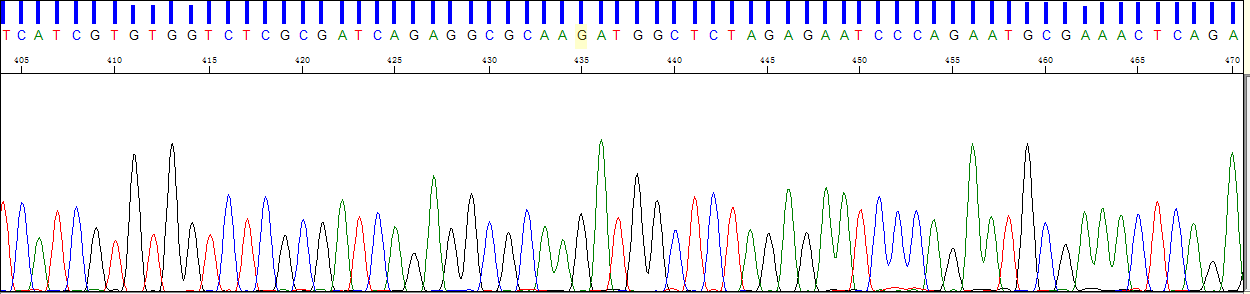


2.DSD09-1,*AR*,c.2158G>A,*Hem*


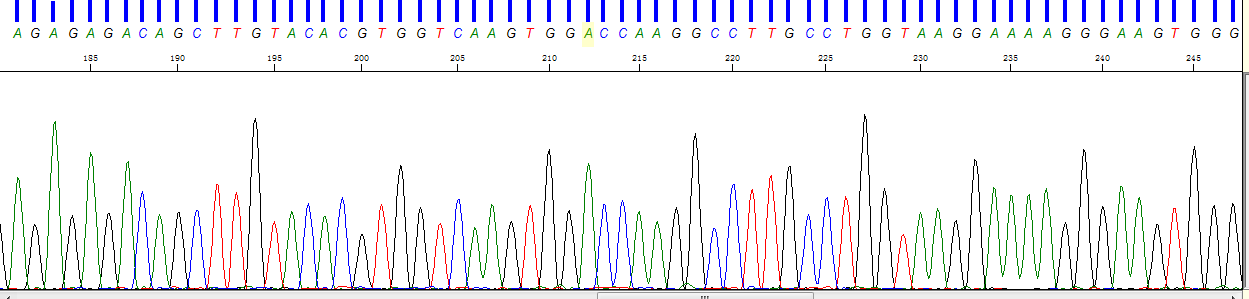


DSD09-2,*AR*,c.2158G>A,*Hem*


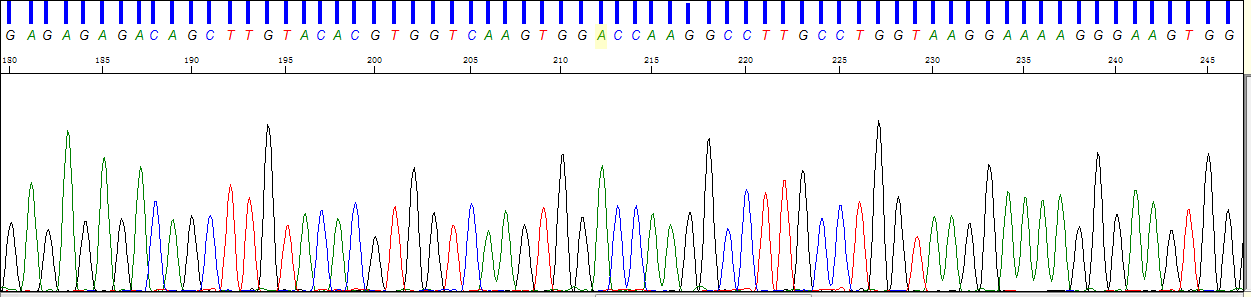


DSD09-Mother,*AR*,c.2158G>A,*Het*


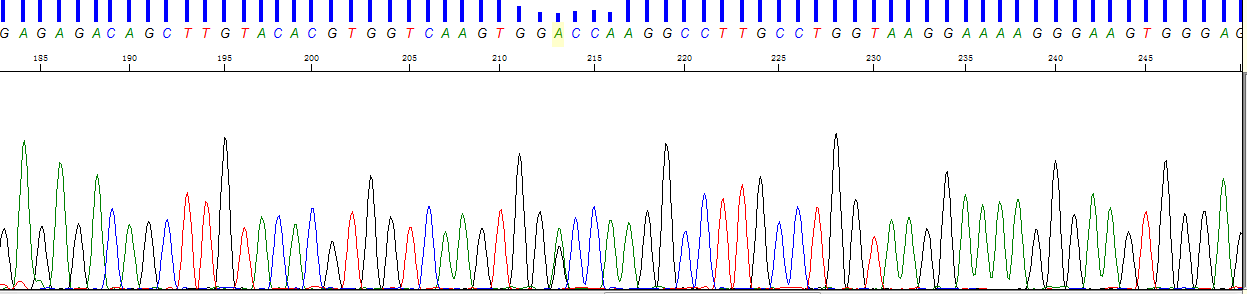


3.DSD18,*AR,*c.1825A>G,*Hem*


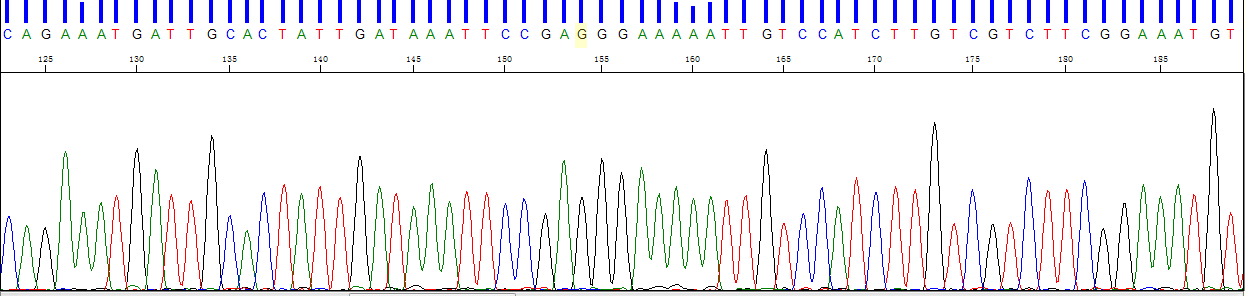


DSD18-Mother,*AR*,c.1825A>G,*Het*


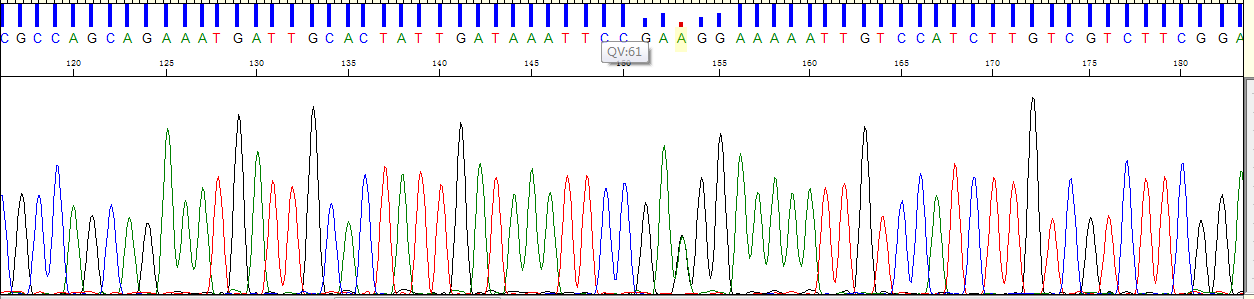


DSD18-Sister, *AR*,c.1825A>G,*Het*


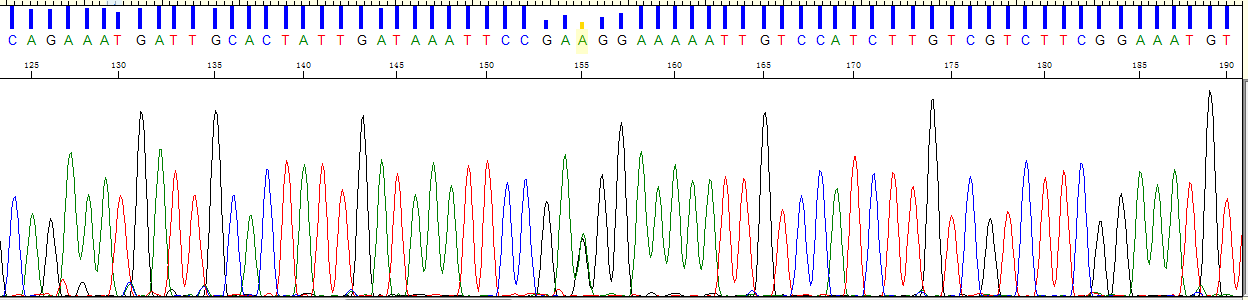


4.DSD20,*AR*,c.2057_2065dupTGTGTGCTG,*Hem*


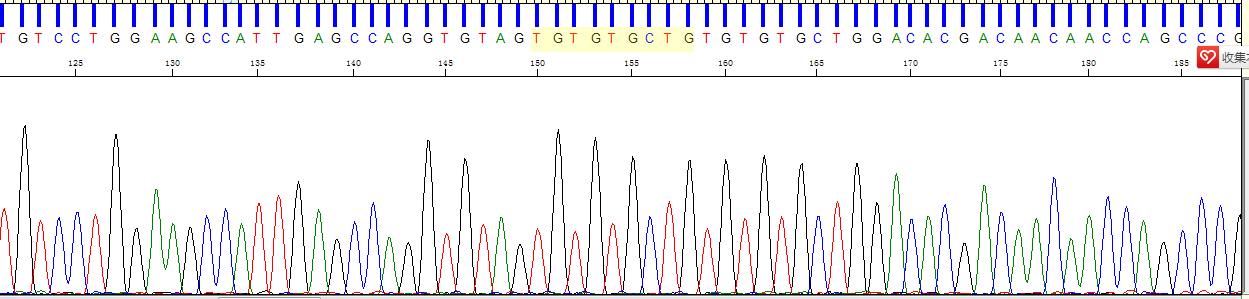


DSD20-Mother,*AR*,c.2057_2065dupTGTGTGCTG,*Het*
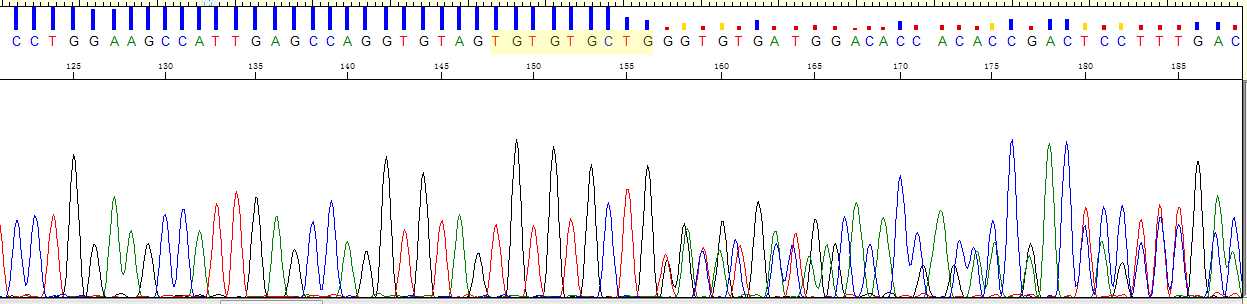


5.DSD04,*CHD7* c.7389delA,*Het*


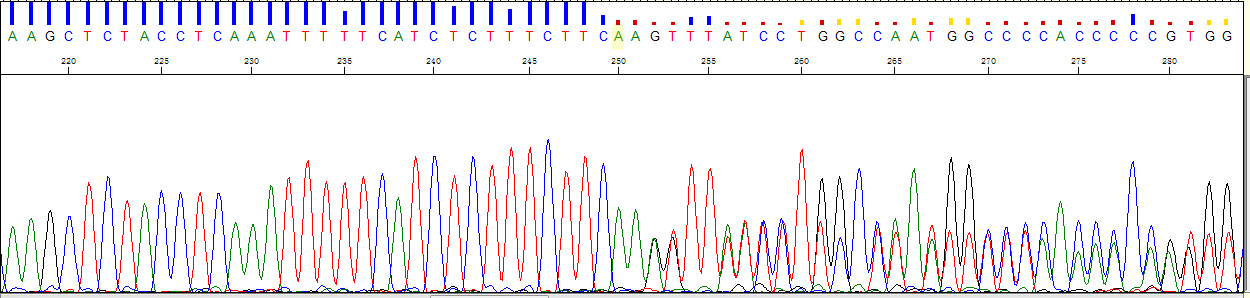


DSD04-father, *CHD7* c.7389delA, normal


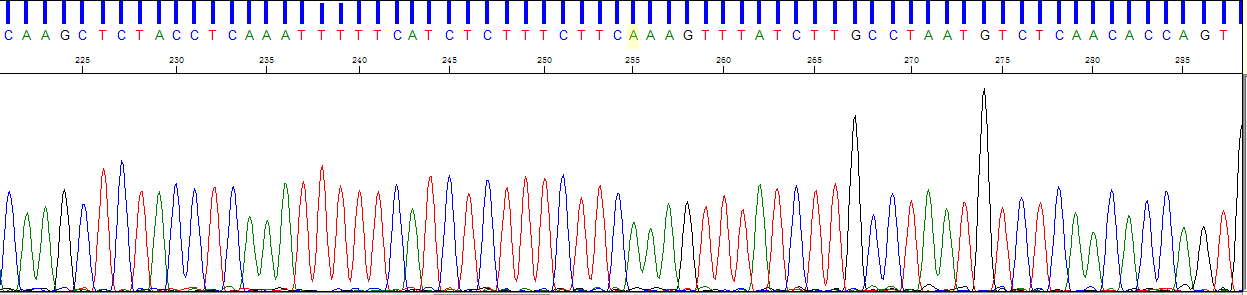


Part2 Sanger sequencing results of samples identified with previous repored mutations

1.DSD07,*NR0B1*,c.273C>G,*Hem*


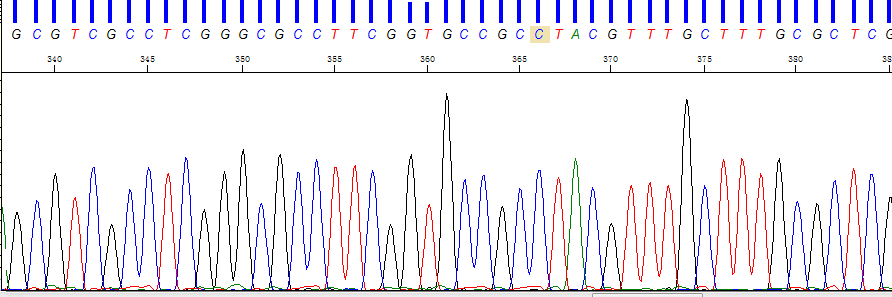


2.DSD13,*CYP17A1*,c.297+2T>C,*Hom*


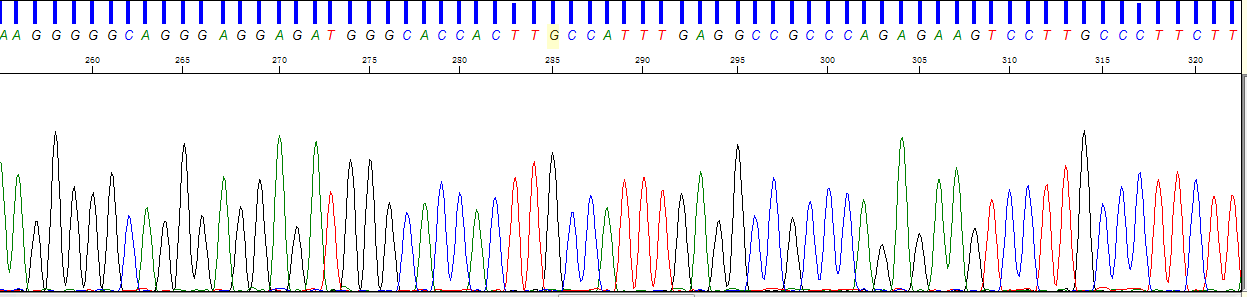


3.DSD14, *AR* ,c.2359C>T,*Hem*


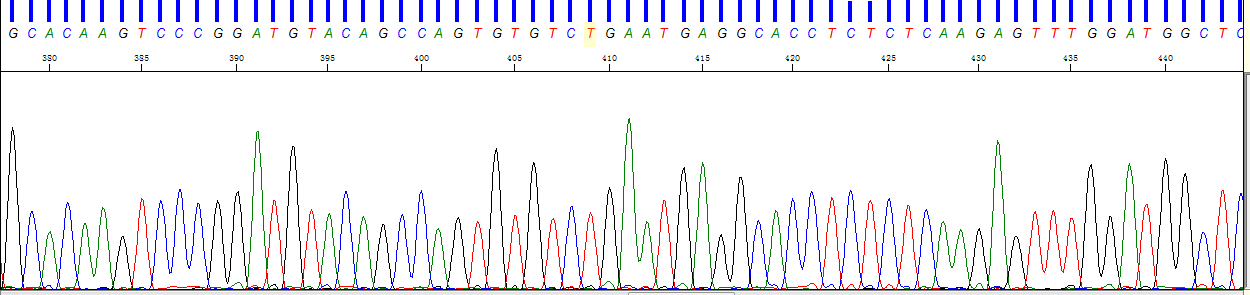


4.DSD17,*AR,*c.174_175insTAGCAGCAGCAGCAG,*Hem*


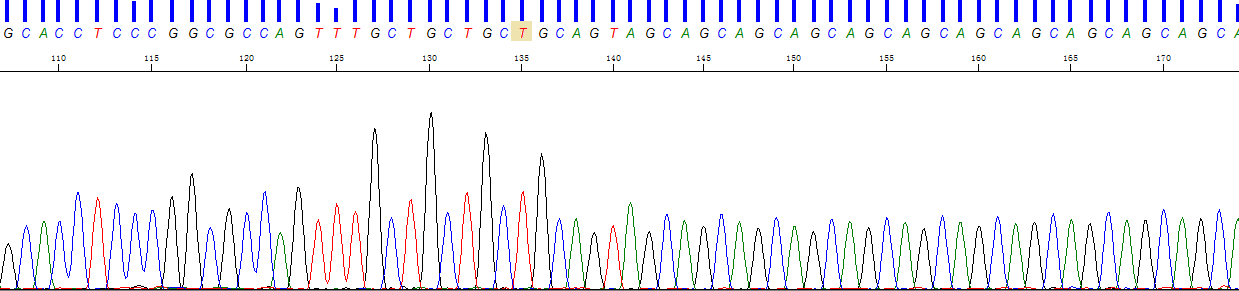


DSD17-Mother,*AR,*c.174_175insTAGCAGCAGCAGCAG,*Het*


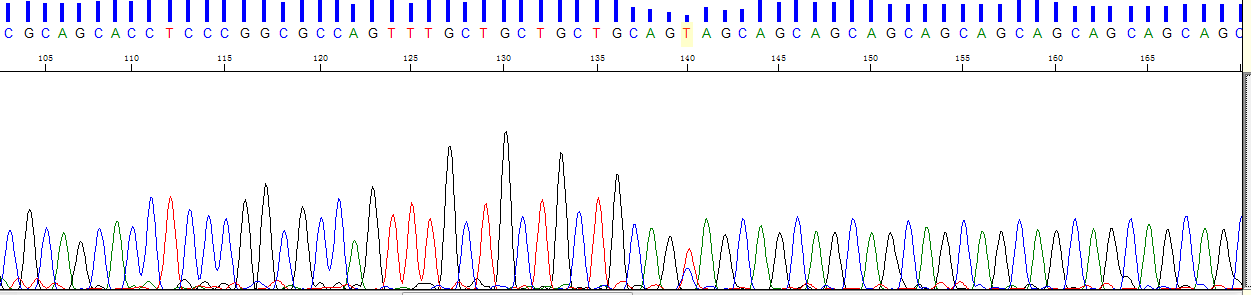


DSD17,*NR5A1*,c.1379A>T,*Het*


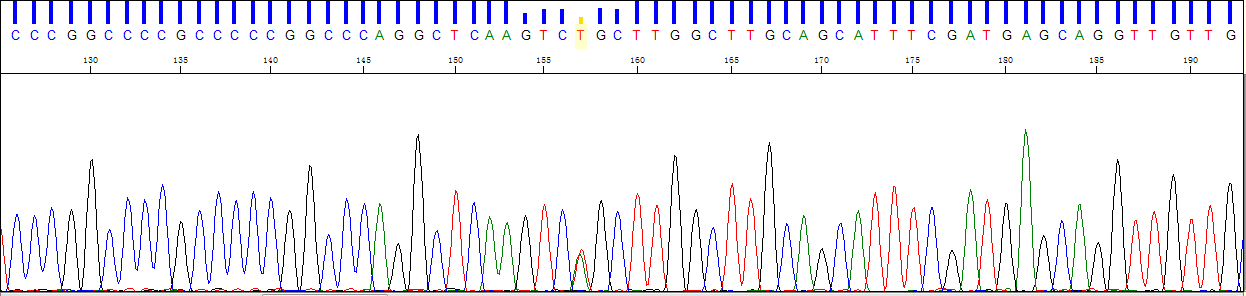


DSD17-Mother,*NR5A1*,c.1379A>T,*Het*


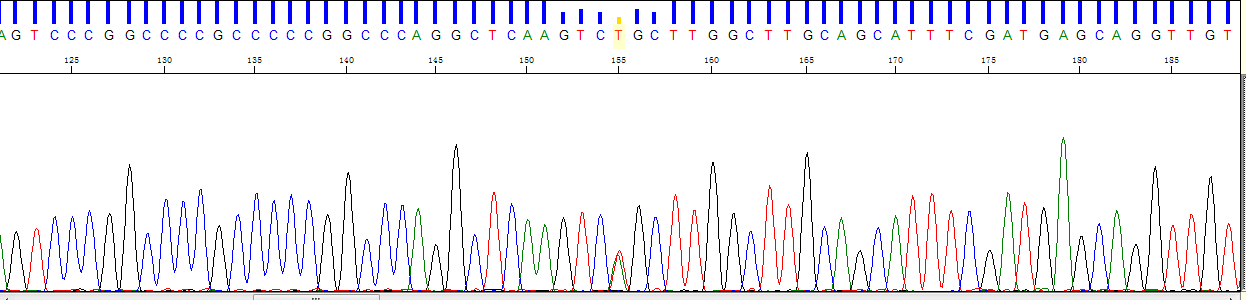


5.DSD21,*SRD5A2*,c.680G>A,*Het*


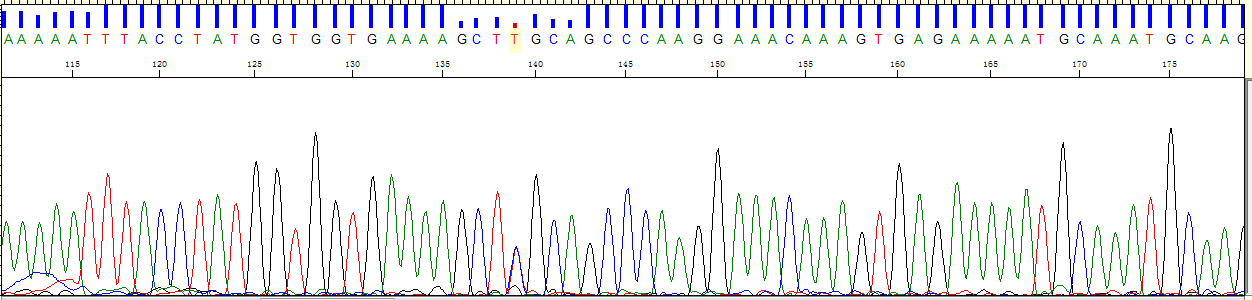


DSD21-Mother,*SRD5A2*,c.680G>A,*Het*


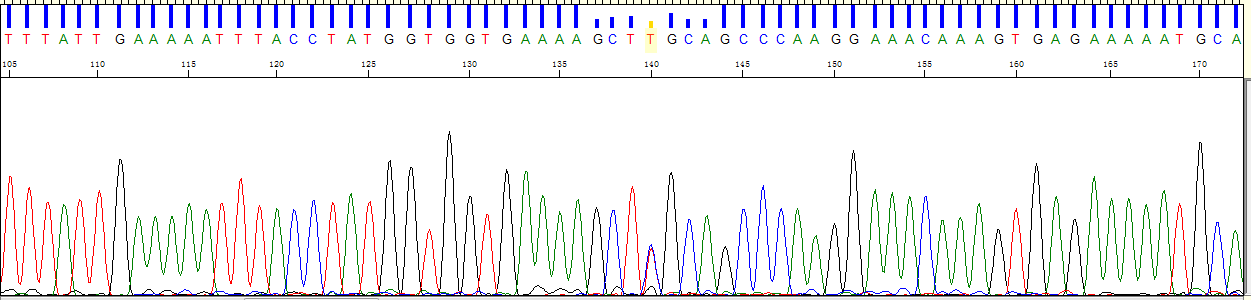

Supplement: Additional file 7: — Sanger sequencing of candidate SNVs and Indels. (DOC 417 kb) [file 12881_2016_286_MOESM7_ESM.doc]
